# Clinical characteristics and serum inflammatory markers of community‐acquired mycoplasma pneumonia in children

**DOI:** 10.1111/crj.13620

**Published:** 2023-05-04

**Authors:** Fei Fan, Jun Lv, Qianyuan Yang, Fei Jiang

**Affiliations:** ^1^ Department of Paediatrics The Affiliated Changzhou No. 2 People's Hospital of Nanjing Medical University Changzhou Jiangsu China

**Keywords:** cytokine, lymphocyte subsets, *Mycoplasma pneumoniae* pneumonia, non‐mycoplasma pneumoniae pneumonia, refractory mycoplasma pneumoniae pneumonia

## Abstract

**Background:**

To compare the demographic and clinical features, laboratory and imaging findings in mycoplasma pneumoniae pneumonia (MPP) children with non‐MPP (NMPP) children and general MPP (GMPP) children with refractory MPP (RMPP) children and analysis the relationship with the severity of disease.

**Methods:**

The study included 265 children with MPP and 230 children with NMPP in the Affiliated Changzhou No. 2 People's Hospital of Nanjing Medical University from 2020 to 2021. The children with MPP included RMPP (*n* = 85) and GMPP (*n* = 180). Demographic and clinical characteristics, laboratory and imaging findings of all children were measured as baseline data within 24 h after admission and the differences between MPP and NMPP, RMPP and GMPP patients were compared. ROC curves were used to evaluate the diagnostic and predictive value of different indicators for RMPP.

**Results:**

Fever duration and hospital stay in children with MPP were longer than those with NMPP. The number of patients with imaging features of pleural effusion, lung consolidation and bronchopneumonia in MPP group was significantly higher than that in NMPP group. Compared with NMPP group, the levels of C‐reactive protein (CRP), procalcitonin (PCT), serum amyloid A (SAA), erythrocyte sedimentation rate (ESR), lactic dehydrogenase (LDH), prothrombin time (PT), fibrinogen (FIB) and D‐dimer and inflammatory cytokines (interleukin [IL]‐6, IL‐8, IL‐10 and IL‐1β) in MPP group were significantly higher (*P* < 0.05). The clinical symptoms and pulmonary imaging findings were more severe in RMPP group. The levels of white blood cell (WBC), CRP, PCT, SAA, ESR, alanine aminotransferase (ALT), LDH, ferritin, PT, FIB, D‐dimer and inflammatory cytokines in RMPP group were higher than those in GMPP group. There was no significant difference in the level of lymphocyte subsets between the RMPP and GMPP group. IL‐6, IL‐10, LDH, PT, D‐dimer and lung consolidation were independent risk factors for RMPP. IL‐6 levels and LDH activity were good predictors of RMPP.

**Conclusion:**

In conclusion, there were differences in clinical characteristics and serum inflammatory markers between MPP group and NMPP group, RMPP group and GMPP group. IL‐6, IL‐10, LDH, PT and D‐dimer can be used as predictive indicators for RMPP.

## Graphical abstract

We compare the demographic and clinical features, laboratory and imaging findings in mycoplasma pneumoniae pneumonia (MPP) children with non‐MPP (NMPP) children and general MPP (GMPP) children with refractory MPP (RMPP) children and analysis the relationship with the severity of disease. This study showed that the measurement of IL‐6, LDH, IL‐10 and D‐dimer can be useful predictors for RMPP. There were differences in clinical characteristics and serum inflammatory markers between MPP group and NMPP group, RMPP group and GMPP group.

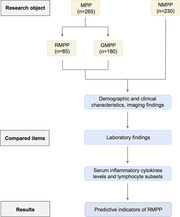

## 1 BACKGROUND

*Mycoplasma pneumoniae* pneumonia (MPP) is an atypical pneumonia characterized by pulmonary interstitial disease that can damage other organs through local respiratory infection. In recent years, due to the influence of environmental changes and other factors, the incidence of MPP has been increasing, accounting for 40% of children's community‐acquired pneumonia (CAP).^1^ The infection rate of MPP in children over 5 years of age could be as high as 50%.^2^ Therefore, MPP was very common in pediatrics. It occurs in all seasons, mainly with subacute onset, and is often manifested by fever, cough, asthma, dyspnoea and other symptoms. Although MPP is a benign and self‐limited disease, some children with MPP may get worse. Severe MPP has a clinical presentation of encephalitis, nephritis, hepatitis or even multiple organ failure, threatening the life of children. The specificity of early lung manifestations in children with MPP is poor, and it is difficult to distinguish it from lung injury caused by other pathogens. This also leads to serious complications. Moreover, with the widespread application of macrolides in children, some children still cannot be effectively treated after 1 week of treatment, resulting in mycoplasma resistance and progressing to refractory mycoplasma pneumoniae pneumonia (RMPP).^3^ RMPP referred to patients with no significant improvement or aggravation or complications of nervous system, cardiovascular system, kidney, gastrointestinal system, skin and blood system after treatment with macrolide antibiotics alone for 1 week.^4^ How to make clear the early diagnosis of MPP and accurately assess its severity is the focus of current pediatric research. It is widely believed that MP proliferates in respiratory epithelial cells by binding P1 protein to cilia, stimulates the production of pro‐inflammatory cytokines in airway mucosa, induces cellular inflammatory response and tissue damage and ultimately leads to changes in host immune function.^5^, ^6^ The imbalance of Th1/Th2 function after *M. pneumoniae* infection is an important immunological mechanism of MPP. Therefore, it is important to study the results of serum inflammatory cytokines in children with MPP for guiding clinical treatment. The purpose of this study was to investigate the differences in clinical manifestations, laboratory tests, levels of inflammatory cytokines and lymphocyte subsets in children with MPP, NMPP and RMPP. We hope to find the predictor of RMPP that can be used to quickly and accurately identify the treatment effect on patients in clinical work and provide a certain basis for the clinical diagnosis and treatment of MPP.

## 2 METHODS

### 2.1 Study subjects

This study is a prospective study; 495 children with pneumonia hospitalized in the Department of Pediatrics of The Affiliated Changzhou No. 2 People's Hospital of Nanjing Medical University from 2020 to 2021 were selected, including 265 children with MPP (including 85 cases of RMPP and 180 cases of GMPP) and 230 cases of NMPP. The inclusion criteria of MPP are as follows: (1) age 1–12 years old; (2) meet the diagnostic criteria of CAP, have respiratory tract infection symptoms, chest imaging findings show pneumonia, with or without pleural effusion; (3) a fourfold or more increase in the serum mycoplasma pneumoniae IgM titer during the recovery period (but not the acute period), or a positive PCR test for mycoplasma pneumoniae in sputum; (4) patients with the concomitant viral infection or specific infection of other pathogens were excluded. The inclusion criteria of NMPP are as follows: (1) age 1–12 years old; (2) meet the diagnostic criteria of CAP; (3) children with confirmed infections of influenza A, influenza B, human metapneumovirus, adenovirus, respiratory syncytial virus, parainfluenza and other viruses were included; (4) children with definite bacterial infection were excluded; (5) children with definite or suspected mycoplasma pneumoniae infection were excluded; (6) children with chlamydia infection were excluded; (7) macrolides and tetracycline antibiotics were not used in this group before and after the admission. Exclusion criteria are as follows: (1) The inclusion criteria were not met, or the clinical data were incomplete; (2) patients who had congenital heart disease, tuberculosis infection, bronchial foreign body, bronchiectasis, metabolic disease, connective tissue disease, inflammatory bowel disease, congenital immune deficiency disease, HIV positive, cerebral palsy or malignant tumor; (3) patients who had a personal or family history of allergies, including asthma, atopic dermatitis and rhinitis; (4) patients who had a history of taking glucocorticoids, bronchodilators or leukotriene receptor antagonists 2 weeks before admission; (5) patients who received mechanical ventilation during treatment; (6) patients who had a history of smoking or passive smoking.

Demographic and clinical features, laboratory and imaging findings of all children included in the study were collected within 24 h after admission, including chief complaint, hospital stays, fever duration, fever, cough, wheezing, chest radiography, lung computed tomography (CT), WBC, CRP, PCT, ESR, neuron‐specific enolase (NSE), ALT, aspartate aminotransferase (AST), albumin, LDH, N‐terminal pro‐B‐type natriuretic peptide (NT‐proBNP) and coagulation indexes. A febrile day was defined as a day on which the body temperature exceeded 37.8°C at least once. The study was approved by the Ethics Committee of The Affiliated Changzhou No. 2 People's Hospital of Nanjing Medical University ([2021]YLJSC012). Informed consent was given in writing by each participant's parents or legal guardian, and this study was conducted in accordance with relevant guidelines and regulations.

### 2.2 Detection of inflammatory factors and lymphocyte subsets

The fasting venous blood (10 mL) was collected from all the children in the next morning after admission, and the serum was separated after centrifugation at 3000 r/min and stored in a −80°C refrigerator. Serum amyloid A (SAA) and serum ferritin (SF) were detected by automatic fluorescence immunoanalyser (IMMULITE 1000, Siemens Ltd). The concentrations of the serum cytokines [IL‐6, IL‐8, IL‐10, IL‐1β, soluble interleukin2 receptor (sIL‐2R), tumor necrosis factor‐α (TNF‐α)] were determined using double‐antibody sandwich enzyme‐linked immunosorbent assay (ELISA, Human Cytokine Panel, Boster Biological Technology Co. Ltd). The percentage of CD3^+^, CD4^+^, CD8^+^, CD16^+^CD56^+^NK and CD19^+^ cells were detected by flow cytometry (FCM, BD FACSCanto II). All experimental steps were strictly in accordance with the instructions. On the morning of the second day after admission, 2 mL of venous blood was taken on an empty stomach, and the blood was centrifuged at 2000 r/min for 10 min, followed by an enzyme‐linked immunosorbent assay (20153400185, Shenzhen YHLO Biotech Co. Ltd) for the detection of mycoplasma pneumoniae IgM antibodies in the children.

### 2.3 Statistical analysis

SPSS software (version 19) was used for statistical analysis. Data distribution in all groups was determined by the Kolmogorov–Smirnov test. The normal distribution data was expressed as mean ± standard deviation (SD). One‐way ANOVA or independent‐samples *T*‐test was used to process these data. Mann–Whitney *U* test was used for comparison between these data. Categorical data were shown as frequencies or percentages. We used Pearson chi‐square test or Fisher's exact test to analyze differences between categorical variables. Stepwise logistic regression model was used for multivariate analysis. Meanwhile, the receiver operating characteristic (ROC) curve was used to evaluate the value of indicators in predicting and diagnosing RMPP. The difference was considered statistically significant at *P* < 0.05.

## 3 RESULTS

### 3.1 Demographic and clinical characteristics, imaging findings of MPP and NMPP children

A total of 495 subjects (265 MPP and 230 NMPP) were enrolled in this study. As shown in Table 1, the median age of children with MPP was markedly older than children with NMPP (4.9 ± 2.5 vs. 3.0 ± 2.1, *P* < 0.001). The days of fever duration and hospital stays in MPP group were longer than those in NMPP group (*P* < 0.001). The number of children with fever (81.5% vs. 73.5%, *P* = 0.03) and extrapulmonary manifestations (34.0% vs. 18.7%, *P* < 0.001) in MPP group were also higher than those in NMPP group. The incidence of pulmonary consolidation (23.0% vs. 15.7%, *P* = 0.04) in children with MPP was higher than those in children with NMPP, while the incidence of bronchopneumonia (31.3% vs. 40%, *P* = 0.04) was lower. There were no significant differences in sex distribution, cough, wheezing, lobar atelectasis, pleural effusion, unilateral lobe pneumonia and bilateral lobe pneumonia between the two groups. The results above indicated that the extrapulmonary manifestations of MPP children were mainly liver function damage, myocardial damage and abnormal coagulation index.

**TABLE 1:** Comparison of demographic and clinical characteristics and imaging findings between MPP and NMPP groups.

General information	MPP(*n* = 265)	NMPP(*n* = 230)	T(Z) or χ^2^	*P* value
Sex (male/female)	120/145	121/109	2.6	0.10
Age (years)	4.9 ± 2.5	3.0 ± 2.1	13.5	<0.001
Fever duration (days)	6 (3,9)	4 (0,6)	−5.5	<0.001
Hospital stay (days)	7 (6,8)	7 (6,7)	−5.3	<0.001
Clinical presentation, *n* (%)				
Fever	216 (81.5%)	169 (73.5%)	4.6	0.03
Cough	265 (100%)	228 (99.1%)	2.3	0.1
Wheezing	15 (5.7%)	12 (5.2%)	0.05	0.8
Extrapulmonary complications	90 (34.0%)	43 (18.7%)	14.6	<0.001
Imaging findings, *n* (%)				
Lobar atelectasis	7 (2.6%)	2 (0.9%)	2.2	0.1
Pleural effusion	6 (2.3%)	1 (0.4%)	3.0	0.09
Lung consolidation	61 (23.0%)	36 (15.6%)	4.2	0.04
Bronchopneumonia	83 (31.3%)	92 (40%)	4.1	0.04
Unilateral lobe pneumonia	93 (35.1%)	65 (28.3%)	2.7	0.1
Bilateral lobe pneumonia	89 (33.6%)	73 (31.7%)	0.2	0.7

*Notes*: The data were the baseline measurement data made within 24 h after admission. *P* < 0.05 indicates statistical significance.

Abbreviations: MPP, mycoplasma pneumoniae pneumonia; NMPP, non‐*Mycoplasma pneumoniae* pneumonia.

### 3.2 Comparison of laboratory findings between the MPP and NMPP groups

The laboratory findings in children with MPP and NMPP were variable and summarized in Table 2. The CRP (11 mg/L vs. 4 mg/L, *P* < 0.001), PCT (0.11 ng/mL vs. 0.09 ng/mL, *P* = 0.01), SAA (50.3 mg/L vs. 25.6 mg/L, *P* < 0.001), ESR (29 mm/h vs. 23 mm/h, *P* < 0.001), LDH (328 U/L vs. 316 U/L, *P* = 0.002), FIB (3.6 g/L vs. 3.2 g/L, *P* < 0.001) and D‐dimer (0.5 mg/L vs. 0.4 mg/L, *P* < 0.001) levels in children with MPP were significantly higher than those in NMPP group, while AST level (29 U/L vs. 32.5 U/L, *P*= 0.004) was lower. But no significant differences were found in WBC, albumin, ALT, SF, NSE and NT‐proBNP between MPP and NMPP groups. In addition, we found that the PT (12.2 s vs. 11.9 s, *P* < 0.001) of children with MPP were significantly longer than that of NMPP children, while the TT (13.7 s vs. 14 s, *P* = 0.002) were markedly shorter.

**TABLE 2:** Comparison of laboratory findings between MPP and NMPP groups.

Laboratory findings	MPP(*n* = 265)	NMPP(*n* = 230)	T(Z) or χ^2^	*P* value
WBC (10^9^/L)	7.7(5.6,10.6)	7.9(5.8,10.5)	−0.3	0.79
CRP (mg/L)	11.0(2.0,25.5)	4.0(1.0,16.0)	−3.9	<0.001
PCT (ng/ml)	0.11(0.06,0.4)	0.09(0.05,0.2)	−2.6	0.01
SAA (mg/L)	50.3(15.3,155.2)	25.6(7.8108.8)	−3.7	<0.001
ESR (mm/h)	29.0(17.0,46.0)	23.0(11.8,35.0)	−3.8	<0.001
ALT (U/L)	16.0(13.0,25.0)	16.0(13.0,20.0)	−1.2	0.2
AST (U/L)	29.0(24.0,37.5)	32.5(27.0,39.0)	−2.9	0.004
Albumin (g/L)	40.5 ± 2.9	41.8 ± 2.9	0.03	0.9
LDH (U/L)	328.0(274.5,404.5)	316.0(272.8,353.5)	−3.1	0.002
SF (ug/L)	117.0(72.1,203.0)	76.1(50.3,122.3)	−6.4	0.07
NSE (mg/mL)	22.6(19.2,26.9)	22.8(19.1,26.4)	−0.05	0.9
NT‐proBNP (pg/mL)	106.9(50.2,192.3)	118.1(58.1,217.9)	−1.7	0.09
APTT (s)	33.0 ± 4.6	32.9 ± 5.1	1.3	0.26
PT (s)	12.2(11.5,13.0)	11.9(11.2,12.5)	−3.7	<0.001
TT (s)	13.7(13.1,14.8)	14.0(13.4,15.1)	−3.1	0.002
FIB (g/L)	3.6(3.0,4.1)	3.2(2.5,3.7)	−4.6	<0.001
D‐dimer (mg/L)	0.5(0.4,1.0)	0.4(0.3,0.6)	−5.3	<0.001

*Notes*: The data were the baseline measurement data made within 24 h after admission. *P* < 0.05 indicates statistical significance.

Abbreviations: ALT, alanine aminotransferase; APTT, activated prothrombin time; AST, aspartate aminotransferase; CRP, C‐reactive protein; ESR, erythrocyte sedimentation rate; FIB, fibrinogen; LDH, lactic dehydrogenase; NSE, neuron‐specific enolase; NT‐proBNP, N‐terminal pro‐B‐type natriuretic peptide; PCT, procalcitonin; PT, prothrombin time; SAA, serum amyloid A; SF, serum ferritin; TT, thrombin time; WBC, white blood cell.

### 3.3 Comparison of the serum inflammatory cytokines levels and lymphocyte subsets between MPP and NMPP groups

The levels of serum inflammatory cytokine in the children with MPP group and NMPP were detected. The result showed that the levels of IL‐6 (7.5 pg/mL vs. 4.9 pg/mL, *P* < 0.001), IL‐8 (39 pg/mL vs. 33.6 pg/mL, *P* = 0.01), IL‐10 (5.7 pg/mL vs. 5 pg/mL, *P* < 0.001) and IL‐1β (11.2 pg/mL vs. 8.2 pg/mL, *P* < 0.001) in the MPP children were significantly higher than those in the NMPP children, but no significant difference in serum sIL‐2R and TNF‐α levels between these two groups. In addition, we also found that the percentages of CD3^+^ (69.2% vs. 65.9%, *P* < 0.001) were remarked increased in children with MPP compared with children with NMPP, while the CD4^+^/CD8^+^ (1.6 vs. 1.7, *P* = 0.01) and the percentages of CD19^+^ (20.2 ± 7.7% vs. 23.1 ± 9.4%, *P* = 0.01) was decreased. No significant differences were found in percentages of CD4^+^, CD8^+^ and CD16^+^CD56^+^NK cell between the two groups (Table 3).

**TABLE 3:** Expression levels of serum inflammatory cytokines and lymphocyte subsets between MPP group and NMPP group.

	MPP(*n* = 265)	NMPP(*n* = 230)	T(Z)or χ^2^	*P* value
IL‐6 (pg/mL)	7.5(3.8,17.0)	4.9(2.7,10.8)	−4.7	<0.001
IL‐8 (pg/mL)	39.0(17.7,100.0)	33.6(16.5,69.6)	−2.5	0.01
IL‐10 (pg/mL)	5.7(5.0,13.6)	5.0(3.3,9.0)	−4.1	<0.001
IL‐1β (pg/mL)	11.2(5.3,19.0)	8.2(4.4,14.8)	−4.0	<0.001
sIL‐2R (U/mL)	970.0(734.0,1257.0)	910.50(705.0,1187.0)	−1.5	0.14
TNF‐α (pg/mL)	17.8(12.4,27.4)	16.8(11.5,23.2)	−1.9	0.06
CD3^+^ (%)	69.2(63.2,73.9)	65.9(60.0,71.1)	−3.5	<0.001
CD4^+^ (%)	36.9 ± 7.7	35.7 ± 8.8	3.0	0.09
CD8^+^ (%)	24.3 ± 6.60	23.7 ± 6.9	1.0	0.32
CD4^+^/CD8^+^ (%)	1.6 ± 0.6	1.7 ± 0.7	7.0	0.01
CD16^+^CD56^+^NK cell (%)	8.7(5.8,13.1)	9.06(5.3,13.2)	−0.2	0.9
CD19^+^ (%)	20.2 ± 7.7	23.1 ± 9.4	6.8	0.01

*Notes*: The data were the baseline measurement data made within 24 h after admission. *P* < 0.05 indicates statistical significance.

Abbreviations: IL‐10, interleukin‐10; IL‐1β, interleukin‐1β; IL‐6, interleukin‐6; IL‐8, interleukin‐8; sIL‐2R, soluble interleukin‐2 receptor; TNF‐α, tumour necrosis factor‐α.

### 3.4 Comparison of demographic and clinical characteristics, imaging findings between RMPP group and GMPP group

We further explored the differences in demographic and clinical characteristics, imaging findings between RMPP and GMPP children. The results were shown in Table 4. There was no statistically significant difference in age and gender between the RMPP and GMPP groups. The RMPP children had a significantly longer fever duration (9 days vs. 7 days, *P* < 0.001) and hospital stays (9 days vs. 7 days, *P* < 0.001) than GMPP children. And the fever (98.8% vs. 73.3%, *P* < 0.001) and extrapulmonary complications (91.8% vs. 6.7% *P* < 0.001) were more frequent in children with RMPP compared to children with GMPP. Meanwhile, significant differences in the incidence rate of pleural effusion (*P* < 0.001), lung consolidation (*P* < 0.001), bronchopneumonia (*P* < 0.001), unilateral lobe pneumonia (*P* < 0.001) and bilateral lobe pneumonia (*P* = 0.04) were observed. The clinical symptoms and pulmonary imaging findings were more severe in RMPP group.

**TABLE 4:** The general characteristics and imaging findings of children with RMPP and GMPP.

General information	RMPP(*n* = 85)	GMPP(*n* = 180)	T(Z) or χ^2^	*P* value
Sex (male/female)	39/46	81/99	0.02	0.9
Age (years)	5.8 ± 2.1	4.4 ± 2.5	3.0	0.08
Fever duration (days)	9.0(8.0,11.0)	4.0(0,7.0)	−9.6	<0.001
Hospital stays (days)	9.0(8.0,10.0)	7.0(6.0,7.0)	−10.8	<0.001
Clinical presentation, *n* (%)				
Fever	84(98.8%)	132(73.3%)	24.9	<0.001
Cough	85(100%)	180(100%)	—	—
Wheezing	2(2.4%)	14(7.8%)	3.00	0.08
Extrapulmonary complications	78(91.8%)	12(6.7%)	186.4	<0.001
Imaging findings, *n* (%)				
Lobar atelectasis	4(4.7%)	3(1.7%)	2.1	0.2
Pleural effusion	6(7.1%)	0	13	<0.001
Lung consolidation	44(51.8%)	17(9.4%)	58.4	<0.001
Bronchopneumonia	4(4.7%)	79(43.9%)	41.2	<0.001
Unilateral lobe pneumonia	45(52.9%)	48(23.9%)	17.5	<0.001
Bilateral lobe pneumonia	36(42.4%)	53(29.4%)	4.3	0.04

*Notes*: The data were the baseline measurement data made within 24 h after admission. *P* < 0.05 indicates statistical significance.

Abbreviations: GMPP, general mycoplasma pneumoniae pneumonia; RMPP, refractory mycoplasma pneumoniae pneumonia.

### 3.5 Comparison of laboratory findings between RMPP group and GMPP group

The detection of the indicator level of RMPP and GMPP children showed that the WBC (9.9 ± 4.4 vs. 7.7 ± 3.6 × 10^9/L, *P* = 0.01), CRP (22 mg/L vs. 7 mg/L, *P* < 0.001), PCT (0.2 ng/mL vs. 0.09 ng/mL, *P* < 0.001), SAA (84.6 mg/L vs. 40.9 mg/L, *P* < 0.001), ESR (36 mm/h vs. 26 mm/h, *P* = 0.01), ALT (31 U/L vs. 15 U/L, *P* < 0.001), LDH (422 U/L vs. 299 U/L, *P* < 0.001) and ferritin (231 ug/L vs. 91.5 ug/L, *P* < 0.001) levels or activities in RMPP group were higher than those in GMPP group with a significant difference. However, the AST, albumin, NSE and NT‐proBNP levels between RMPP and GMPP group were not statistically difference. Similarly, we also compared the blood coagulation indexes between these two groups. The PT (12.6 s vs. 12 s, *P* < 0.001), FIB (3.9 g/L vs. 3.3 g/L, *P* < 0.001) and D‐dimer (1.1 mg/L vs. 0.5 mg/L, *P* < 0.001) levels in RMPP children were significantly increased compared with GMPP children. However, we did not find significant differences in APTT and TT between groups (Table 5).

**TABLE 5:** The laboratory test index of children with RMPP and GMPP.

	RMPP(n = 85)	GMPP(n = 180)	T(Z)or χ^2^	*P* value
WBC (10^9^/L)	9.9 ± 4.4	7.7 ± 3.6	6.4	0.01
CRP (mg/L)	22.0(8.5,38.5)	7.0(1.3,19,8)	−5.6	<0.001
PCT (ng/ml)	0.2(0.1,1.2)	0.09(0.05,0.2)	−5.3	<0.001
SAA (mg/L)	84.6(29.1192.2)	40.9(8.8116,8)	−3.9	<0.001
ESR (mm/h)	36.0(25.0,52.5)	26.0(15.0,43.0)	−2.5	0.01
ALT (U/L)	31.0(15.0,43.0)	15.0(12.0,19.0)	−5.5	<0.001
AST (U/L)	31.0(25.0,41.5)	29.0(24.0,35.0)	−1.7	0.08
Albumin (g/L)	39.5 ± 3.1	41.0 ± 2.8	0.7	0.39
LDH (U/L)	422.0(342.5527.5)	299.00(266.0,352.0)	−8.3	<0.001
Ferritin (ug/L)	231.0(130.0,339.1)	91.5(58.2,139.3)	−8.3	<0.001
NSE (mg/ml)	23.0(19.2,28.05)	22.5(19.2,25.9)	−0.8	0.4
NT‐proBNP (pg/mL)	118.7(61.7,239.6)	100.5(44.3,184.0)	−1.94	0.05
APTT (s)	33.5 ± 5.9	32.8 ± 3.9	2.6	0.1
PT (s)	12.6(11.8,13.6)	12.0(11.4,12.7)	−4.6	<0.001
TT (s)	13.5(12.9,15.0)	13.8(13.2,14.8)	−1.2	0.2
FIB (g/L)	3.9(3.3,4.4)	3.3(2.8,4.0)	−4.4	<0.001
D‐dimer (mg/L)	1.1(0.7,1.7)	0.45(0.35,0.7)	−8.2	<0.001

*Notes*: The data were the baseline measurement data made within 24 h after admission. *P* < 0.05 indicates statistical significance.

Abbreviations: ALT, alanine aminotransferase; APTT, activated prothrombin time; AST, aspartate aminotransferase; CRP, C‐reactive protein; ESR, erythrocyte sedimentation rate; FIB, fibrinogen; LDH, lactic dehydrogenase; NSE, neuron‐specific enolase; NT‐proBNP, N‐terminal pro‐B‐type natriuretic peptide; PCT, procalcitonin; PT, prothrombin time; SAA, serum amyloid A; SF, serum ferritin; TT, thrombin time; WBC, white blood cell.

### 3.6 Comparison of serum inflammatory cytokines and lymphocyte subsets between RMPP group and GMPP group

As shown in Table 6, there were no significant differences in the lymphocyte subsets and TNF‐α levels between these two groups. But serum IL‐6 (18 pg/mL vs. 5.7 pg/mL, *P* < 0.001), IL‐8 (92.5 pg/mL vs. 27.3 pg/mL, *P* < 0.001), IL‐10 (11.3 pg/mL vs. 5 pg/mL, *P* < 0.001), IL‐1β (16.8 pg/mL vs. 8.3 pg/mL, *P* < 0.001) and sIL‐2R (1068 pg/mL vs. 849.5 pg/mL, *P* < 0.001) levels were significantly higher in the RMPP group compared with the GMPP group.

**TABLE 6:** Expression levels of serum inflammatory cytokines and lymphocyte subsets between RMPP group and GMPP group.

	RMPP(*n* = 85)	GMPP(*n* = 180)	T(Z)	*P* value
IL‐6 (pg/mL)	18.0(10.4,29.6)	5.74(3.2,10.2)	−8.0	<0.001
IL‐8 (pg/mL)	92.5(27.9,248.6)	27.30(15.9,79.4)	−5.7	<0.001
IL‐10 (pg/mL)	11.3(5.7,27.6)	5.0(4.4,9.0)	−6.2	<0.001
IL‐1β (pg/mL)	16.8(10.4,32.7)	8.3(5.0,15.0)	−6.7	<0.001
sIL‐2R (U/mL)	1068.0(909.0,1565.5)	849.5(689.3,1202.3)	−4.5	<0.001
TNF‐α (pg/mL)	19.3(14.0,28.8)	17.1(12.0,26.6)	−1.8	0.07
CD3^+^ (%)	67.6(59.9,73.6)	70.0(64.2,74.1)	−1.4	0.2
CD4^+^ (%)	36.3 ± 8.2	37.25 ± 7.5	2.1	0.2
CD8^+^ (%)	24.0 ± 6.4	24.4 ± 6.7	0.2	0.7
CD4^+^/CD8^+^ (%)	1.6 ± 0.6	1.7 ± 0.6	0.2	0.7
CD16^+^CD56^+^NK cell (%)	9.8(6.4,14.9)	8.2(5.4,12.4)	−1.9	0.05
CD19^+^ (%)	20.3 ± 8.2	20.2 ± 7.5	0.1	0.7

### 3.7 Correlation between different indicators and RMPP

We performed logistic regression analysis of serum inflammatory cytokines, laboratory indicators and imaging indicators with significant differences between RMPP group and GMPP group based on all baseline data of children with MPP. The IL‐8, IL‐1β, sIL‐2R, WBC, CRP, PCT, SAA, ESR, ALT, ferritin, FIB and pleural effusion were excluded from the analysis due to their high non‐response rate. The logistic regression results shown that the IL‐6 (*P* = 0.03), IL‐10 (*P* = 0.02), LDH (*P* < 0.001), PT, D‐dimer (*P* < 0.001) and lung consolidation (*P* < 0.001) were independent risk factors for RMPP, so they were included as the predictive indicators (Table 7).

**TABLE 7:** Logistic regression analysis predictors of RMPP.

Variable	β	SE	Wald	*P* value	Exp(B)	95% CI	
IL‐6	−0.04	0.02	4.8	0.03	1.0	0.9	1.0
IL‐10	−0.04	0.02	5.5	0.02	1.0	0.9	0.99
LDH	−0.01	0.002	15.3	<0.001	1.0	0.99	0.995
PT	−0.6	0.2	10.6	0.001	0.6	0.4	0.8
D‐dimer	−1.1	0.4	8.83	<0.001	0.3	0.2	0.7
Lung consolidation	−2.9	0.5	36.0	<0.001	0.05	0.02	0.14
WBC	−0.01	0.05	0.04	0.8	1.0	0.9	1.1
CRP	−0.002	0.01	0.05	0.8	1.0	0.98	1.0
PCT	−0.3	0.3	1.3	0.3	0.7	0.4	1.3
SAA	0.003	0.002	1.6	0.2	1.0	1.0	1.0
ESR	0.01	0.01	1.0	0.3	1.0	0.99	1.0
ALT	0.02	0.01	3.9	0.05	1.0	1.0	1.1
Ferritin	0.004	0.002	4.0	0.05	1.0	1.0	1.0
FIB	0.4	0.2	3.7	0.05	1.6	1.0	2.4
IL‐8	0.002	0.001	3.5	0.06	1.0	1.0	1.0
IL‐1β	0.001	0.01	0.03	0.9	1.0	0.99	1.0
Pleural effusion	22.0	20096.5	0	1.0	0	0	

Abbreviation: SE, standard error.

### 3.8 Predictive value of independent correlation factors for RMPP

**FIGURE 1:**
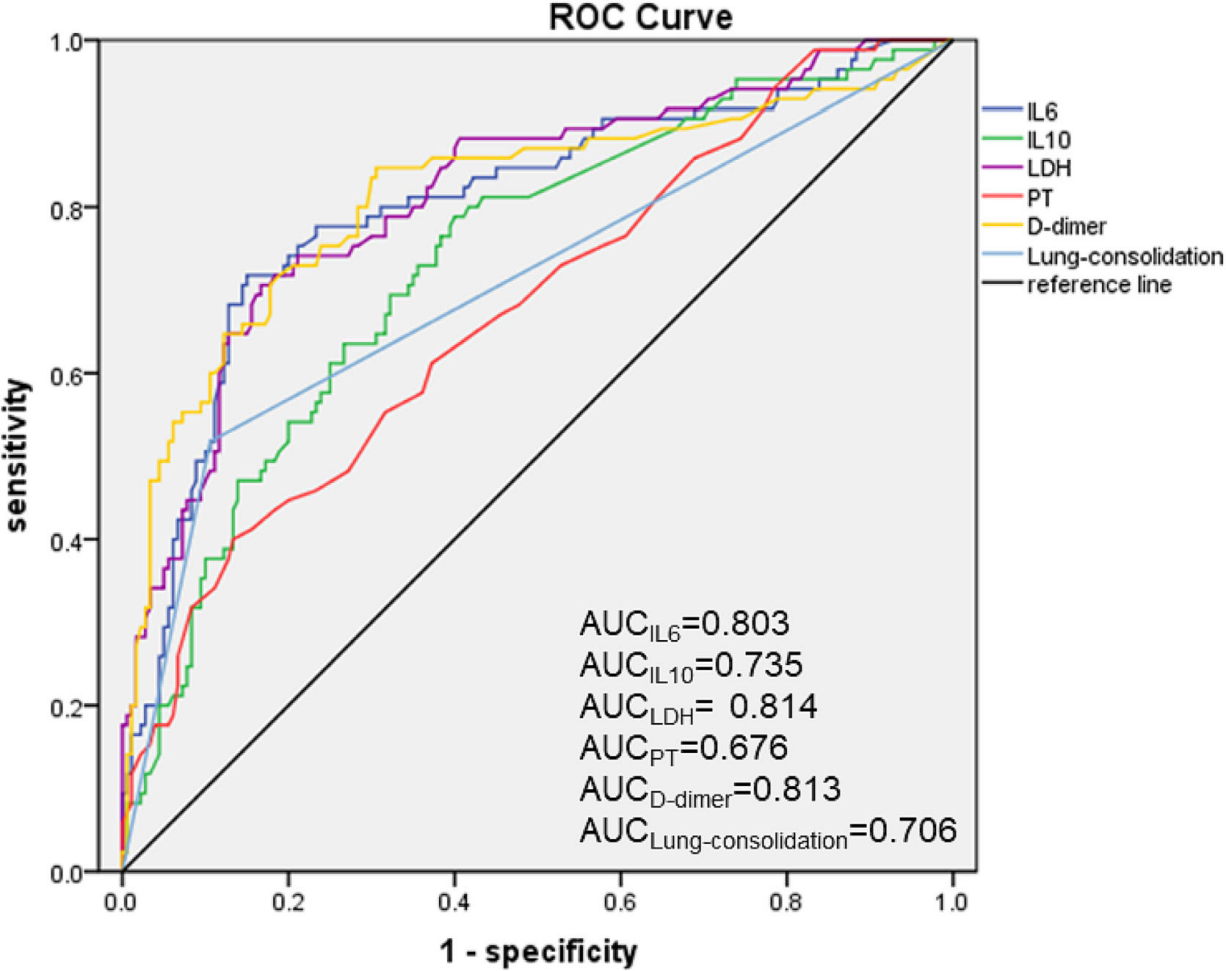
ROC curves of IL‐6, IL‐10, LDH, PT, D‐dimer and lung consolidation for predicting RMPP.

The results of ROC curves showed that the IL‐6 level (AUC = 0.803, sensitivity = 70%, specificity = 80%) above 13.2 pg/mL and LDH activity (AUC = 0.814, sensitivity = 70%, specificity = 80%) above 378 U/L were good predictors for RMPP. An IL‐10 level of 5.6 pg/mL was the second most useful biomarker, with an AUC of 0.735, a sensitivity of 80% and a specificity of 60%. Lung consolidation had a predictive value in the RMPP, but its sensitivity of 50% was low. Although D‐dimer had a high AUC of 0.813, its specificity of 70% was low. PT had the least predictive value for RMPP (Table 8 and Figure 1).

**TABLE 8:** Predictive value of independent correlation factors.

Independent factors	Cut‐off value	AUC	SE	95%CI		Sensitivity	Specificity	*P* value
IL‐6	13.2	0.803	0.03	0.7	0.9	0.7	0.8	<0.001
IL‐10	5.6	0.735	0.03	0.7	0.8	0.8	0.6	<0.001
LDH	378.0	0.814	0.03	0.757	0.9	0.7	0.8	<0.001
PT	13.2	0.676	0.03	0.6	0.7	0.4	0.9	<0.001
D‐dimer	0.6	0.813	0.03	0.8	0.9	0.8	0.7	<0.001
Lung consolidation	1	0.706	0.037	0.5	0.8	0.5	0.9	<0.001

*Note*: 1 = lung consolidation; 0 = no lung consolidation.

Abbreviations: AUC, area under curve; SE, standard error.

## 4 DISCUSSION

MPP is a common pathogen of CAP in children. In our study, there were 85 children with RMPP, accounting for 32.08% of MPP group. According to the statistical results, the children in MPP group were older than those in NMPP group. It was found that fever and extrapulmonary manifestations were more frequent in children with MPP. Some studies had shown that MPP had various extrapulmonary manifestations including meningitis, cerebral infarction, myocarditis, hepatitis and so on.^7^, ^8^ The extrapulmonary manifestations were mainly liver function damage, myocardial damage and abnormal coagulation index in our research. More serious extrapulmonary complications were not found, which might be due to the small number of MPP patients enrolled.

MP can infect the entire airway, even the interstitial lung and alveoli. So the imaging findings of MPP may vary depending on the region of MP infection. From the perspective of chest imaging, there was little difference in the sites of the two types of pneumonia (MPP and NMPP). However, in our study, the incidence of pulmonary consolidation in children with MPP group was higher than those in NMPP group. In a previous study with 393 hospitalized children diagnosed with MMP, the most common radiological finding was lobar or segmental consolidation (37%).^9^ Therefore, MP infection is more likely to cause the development of lung tissue damage. The severity of MPP should be identified as soon as possible in clinical work to prevent adverse results.

White blood cell (WBC), C‐reactive protein (CRP), procalcitonin (PCT), serum amyloid A (SAA) and erythrocyte sedimentation rate (ESR) are highly sensitive markers for infection and inflammation. When compared with the NMPP group, the CRP, PCT, SAA, ESR and LDH levels in MPP children were significantly higher. Some researchers also found that SAA, CRP and PCT were specific markers for diagnosing early MP infection in children.^10^ Although some differences were obtained, these indicators could not be used to distinguish MPP from NMPP due to their low specificity. These specific markers need to be considered in combination with other cytokines and clinical characteristics. MP infection activates the exogenous and endogenous coagulation system through a variety of ways, leading to coagulation abnormalities and promotion of thrombosis.^11^ In the indices of blood coagulation, we found that MPP children had higher levels of prothrombin time (PT), thrombin time (TT), fibrinogen (FIB), D‐dimer and a lower level of TT than NMPP children. This result indicated that children with MPP had a higher risk of embolism.

Cellular immunity plays an important role in the development of MPP. CD8 + T cells inhibit the pulmonary immune response, while CD4 + T cells were related to the severity of MP.^12^ Our study had shown that there were significant differences in the percentages of CD3^+^, CD4^+^/CD8^+^ and CD19^+^ between the MPP group and NMPP group. The mechanism of our conclusion is unclear. It is possible that the patients with NMPP we enrolled were related to cellular immune disorders. So further large‐scale studies are needed.

Some researchers had found that the cytokine‐mediated inflammation and immune response of the host played an important role in mycoplasmal pathogenicity.^13^ In this study, we analyzed the levels of serum IL‐6, IL‐8, IL‐10, IL‐1β, sIL‐2R and TNF‐α in children with pneumonia. The expression levels of serum IL‐6, IL‐8, IL‐10 and IL‐1β in the MPP children were significantly higher than those in the NMPP children. In addition, sIL‐2R was also analyzed. sIL‐2R is a soluble mucin that can prolong the half‐life of IL‐2 and aggravate inflammatory injury. Our analysis had shown that no significant difference was observed in serum sIL‐2R level between MPP group and NMPP group, which has not been reported so far by other teams.

This study was also designed to understand the characteristics of RMPP. So, we compared the clinical manifestations, imaging manifestations and laboratory indicators of the RMPP group and the GMPP group. It had been confirmed that patients with RMPP had a significantly longer duration of fever, length of hospitalization and higher incidence of extrapulmonary complications than those with non‐RMPP, which was consistent with our research results.^12^, ^14^ Chest radiography and CT at admission showed that the incidence rate of lung consolidation was higher in the RMMP group than in the GMMP group. The pulmonary imaging findings were more severe in RMPP group. In addition, the proportion of those with pleural effusion was higher in the RMMP group. This is consistent with previous reports.^15^, ^16^

Prediction of RMPP at the early stages of MP is crucial. In this study, several serum markers were tested to assess whether they could predict RMMP. As in previous reports,^15^, ^17^, ^18^ CRP, ESR, ALT and LDH levels in RMPP group were higher, with a significant difference. Choi et al^15^ found that the leukocyte count was within the normal range in all MPP groups. But we found that leukocyte count in RMPP group was higher than that in GMPP group. It may be associated with a stronger immune response in RMPP group. PCT, SAA and ferritin levels are also closely related to the inflammatory response. It has been reported that serum ferritin level may be useful as an indicator of the severity of pediatric MPP for initiation of corticosteroid therapy.^19^ PCT, SAA and ferritin were also significantly increased in the RMPP patients. Therefore, these significant inflammatory markers may be predictors of RMPP. The RMPP group showed higher levels of serum IL‐6, IL‐8, IL‐10, IL‐1β and sIL‐2R than the GMPP group. The logistic regression analysis confirmed that IL‐6, IL‐10, LDH, PT, D‐dimer and lung consolidation were independent risk factors for RMPP. According to the results of ROC curves, it showed that IL‐6 ≥ 13.2 pg/mL and LDH ≥ 378 U/L were good predictors for RMPP. Guo et al^20^ had reported elevated levels of IL6 in children with RMPP compared to control. It was found that IL‐6 ≥ 14.75 pg/mL was a significant predictor regarding RMPP.^21^, ^22^ In future clinical work, we need to further verify our research results.

In summary, we found that children with MPP were more likely to have fever, extrapulmonary complications, serious lung imaging changes, abnormal coagulation function and higher inflammatory markers (CRP, PCT, SAA, ESR, LDH, IL‐6, IL‐8, IL‐10 and IL‐1β). In clinical work, if similar manifestations are found in children, the possibility of MP infection should be considered, and macrolide antibiotics should be used as soon as possible. Meanwhile, this study has shown that the measurement of IL‐6, LDH, IL‐10 and D‐dimer can be useful predictors for RMPP. In patients with MP, if lung consolidation is found on chest imaging and high levels of IL‐6, IL‐10, LDH and D‐dimer are detected on laboratory tests at the beginning of hospitalization, an impending refractory reaction to macrolides should be considered. In such situations, glucocorticoids should be used as early as possible. Compared with other studies, these indicators included in our study are easy to obtain and more conducive to full use in clinical work.

Our study has the following limitations. This was a single‐centre study, and the number of patients enrolled in the RMPP group was relatively small. Some patients included in the study might have co‐infection with other pathogens that were not detected during hospitalization. The severity of patients' disease was not evenly distributed, which had a certain impact on the experimental results. All children are Han and the source is relatively single. The results of this study may not be suitable for patients of other ethnic groups or races. Meanwhile, the blood samples collected for analysis were not controlled at a uniform point in time. In addition, predictors of RMPP were identified based on data from all children with MPP; we did not have sufficient data to divide it into learning and test sets; C statistics calculated from a test set may have been lower than those reported here. These impact factors may lead to some research bias and should be further confirmed in larger, multi‐centre, prospective studies in the future.

In conclusion, we found significant differences in clinical features, imaging findings and serum inflammatory markers of MPP. This single‐centre study confirmed that the measurement of IL‐6, LDH, IL‐10 and D‐dimer can be useful markers for RMPP. This study may help paediatricians to estimate early identification of MP infection and assess its severity. Further studies conducted in multiple centres are needed to validate our research.

## AUTHOR CONTRIBUTIONS

Fei Fan and Qianyuan Yang conceptualized and designed the study, carried out the initial analyses. Jun Lv and Fei Jiang drafted the initial manuscript and reviewed and revised the manuscript; all authors approved the final manuscript as submitted.

## CONFLICT OF INTEREST STATEMENT

The authors declare that they have no competing interests.

## ETHICS STATEMENT

The study was approved by the Ethics Committee of The Affiliated Changzhou No. 2 People's Hospital of Nanjing Medical University ([2021]YLJSC012). Informed consent was given in writing by each participant's parents or legal guardian, and this study was conducted in accordance with relevant guidelines and regulations.

## Data Availability

The datasets used and/or analysed during the current study are available from the corresponding author on reasonable request.
